# Use of continuous subcutaneous anesthetic infusion in cardiac surgical patients after median sternotomy

**DOI:** 10.1186/1749-8090-3-2

**Published:** 2008-01-25

**Authors:** Ioannis Koukis, Mihalis Argiriou, Antonia Dimakopoulou, Victor Panagiotakopoulos, Nikolaos Theakos, Christos Charitos

**Affiliations:** 1 Department of Cardiac Surgery, Evangelismos General Hospital, Athens, Greece; 2 Department of Anesthesiology, G. Gennimatas General Hospital, Athens, Greece

## Abstract

The use of opioid analgesics to control pain after median sternotomy in cardiac surgical patients is worldwide accepted and established. However, opioids have a wide range of possible side effects, concerning prolonged extubation time, gastrointestinal tract dyskinesia and urinary tract disorders mostly retention. All these may lead to a prolonged ICU stay or overall hospitalization time increase.

To determine whether a continuous subcutaneous regional anesthetic infusion delivered directly to the sternotomy site would result in decreased levels of postoperative pain and opioid requirements in cardiac surgical patients undergoing median sternotomy.

The continuous subcutaneous infusion (OnQ Painbuster system) was applied in 37 patients. 3 patients were exempted due to prolonged ICU stay. 29 patients underwent CABG, 5 had AVR, 1 MVR and modified Maze, 1 patient had a 3-valve repair due to endocarditis and another one had reconstruction of the left ventricle. Requirements of opioid analgesics were recorded for 96 hours after operation. Pain was assessed using the visual analog scale and the total postoperative hospital length of stay was also measured.

The postoperative pain was significantly diminished (0 – 3 at VAS). The mean postoperative length of stay was 5,8 days, rather improved compared to the average stay of 6,7 days.

Continuous subcutaneous infusion of ropivacaine directly at the median sternotomy significantly diminishes postoperative pain and the need for opioid analgesic use. Moreover, it seems to reduce overall postoperative length of stay for all cardiac surgical patients.

## Background

One of the majors concerns of any surgical patient entering an ICU is pain. In cardiac surgical patients, pain is mostly due to median sternotomy (or sometimes thoracotomy) and it seems to be higher on the first two days which is the usual length of stay at the cardiac ICU [1]. Traditional teaching suggests that pain after median sternotomy is alleviated best with parenteral opioid-based analgesics. Non-steroid anti-inflammatory agents (NSAIDS) or other types of analgesics failed to show significant benefit over opioids in cardiac surgical patients. However, opioid analgesics are well known for their major and sometimes dreadful side effects, especially on cardiac surgical patients who should be discharged from ICU on a fast tract protocol [1-3]. Opioid adverse effects such as respiratory depression, nausea, vomiting, decreased gastro-intestinal (GI) motility or even ileus and urinary retention can prolong the length of stay of patients on the cardiac surgical ICU or at the ward. This undesired prolongation of length of hospital stay because of opioid side effects is well known to surgeons, anesthesiologists and nursing personnel caring for the cardiac surgical patient sustaining a median sternotomy [2,4].

There are several methods to deliver opioid analgesia, mostly patient controlled or nurse controlled. None of them seem to be free of opioid adverse effects, though. This is especially true in cardiac surgical patients after median sternotomy. These patients need to remain calm and relaxed. Most important, they should be able to take easy, deep breaths to avoid atelectasis and get out of bed as soon as possible. All these cannot happen when they are in pain [5].

Multimodal analgesia, combining the use of nerve blockade followed by intravenous analgesia in the usual manner has been tried but failed to show any particular benefit in clinical trials [2,4,6]. The need to find a way to safely and easily reduce pain in cardiac surgical patients sustaining medial sternotomy is more than obvious. More that 10,000 median sternotomies for coronary arterial by-pass grafting (CABG), valve or aortic operations are performed annually in Greece. Improved pain control and subsequent improved patient outcomes would provide a substantial national health care benefit. Therefore, we designed this clinical study to examine the hypothesis that the continuous subcutaneous infusion of a local anesthetic non-opioid solution at the median sternotomy site would produce sufficient pain control so as to completely or at least partly avoid the use of intravenous opioid analgesics in cardiac surgical patients. The overall hospital length of stay was also examined.

## Materials and methods

The study population included 76 adult patients. They all underwent median sternotomy. The various cardiac operations included CABG (43 patients), AVR (17 patients), MVR (9 patients), MVR and ablation for atrial fibrillation (AF) (5 patients), triple valve operation for endocarditis (1 patient) and reconstruction of an aneurysm of the left ventricle – Dor operation (1 patient). The exclusion criteria were prolonged ICU stay more than three days, failure of the patients to comprehend and cooperate with the visual analog pain scale (VAS) preoperatively and failure to understand and sign the appropriate informed consent. The demographic characteristics of the patients and operative management are summarized on table 1.

**Table 1:** Demographic characteristics and operative management of study group

	Ropivacaine study group
Number of patients	76
Age (mean)	69
Sex (m/f)	54/22
CABG	43
AVR	17
MVR	9
MVR + ablation	5
Triple-valve	1
Aneurysm of left ventricle	1

Each of the patients was given a thorough explanation of the study by one of the surgeons in our department. Then, the patient was introduced to the VAS. This was our choice to try to calculate and rate the pain derived from the median sternotomy. Patients were taught to use the colors on the scale and match them with the severity of pain. The scale ranged from 0 (no pain) to 10 (maximum pain imaginable). Study participants were informed that they could use intravenous drugs to alleviate severe postoperative pain but they should do so only when the pain was really disturbing and severe. This study did not incur any additional cost to the patient. The cost of the cardiac operation was fully covered by the patients' insurance organizations, as usual.

Cardiac operations were performed in the usual manner and at the end of the operation the sternum was reapproximated with heavy wire. Exactly above the sternum and before closing the subcutaneous fascia, two especially designed catheters were placed on the sternum, through the skin using matching introduction hollow needles and sheaths. These slim catheters were equipped with multiple small side openings towards their end. The drastic length of each catheter, the part with the holes was 12,5 cm (4,92 in). They were placed right above the wired sternum and stabilized by sticking the tip of the catheter under the first wire. This method of stabilization was developed by our group with intend to avoid placing extra absorbable sutures to stabilize the catheters. It also helped in removal of them. We did not experience any catheter breaks on removal, as noted by other groups [1]. The catheters were connected to a pressurized elastomeric pump equipped with a standard flow regulator which allowed for delivery of the anesthetic mixture at a fixed rate of 4 ml/h. the volume of the pump was 400 ml. The pump was filled with a mixture containing 300 ml ropivacaine (2 mg/ml) plus 100 ml ropivacaine (7,5 mg/ml). The catheters were not stitched to the patients' skin to avoid patients' discomfort. Instead they were stabilized with an adhesive tape on the skin, next to the mediastinal drains. The whole specially designed system which was used was the OnQ Painbuster pain relief system provided by CORMED Hellas.

The choice of the proper anesthetic to use was between two long acting local anesthetic agents, namely ropivacaine and bupivacaine. Literature suggests though that ropivacaine sustains significantly less cardiotoxicity than bupivacaine [1,3]. For that reason, ropivacaine was our local anesthetic of choice.

The catheters were removed after four days. Nurses at the ICU and the ward were supplied with a form for each patient. In that form they filled the number of times they used intravenous opioids to comfort the patients during the first four postoperative days and the dosage given. They also marked the time passed to extubation, the time until the first bowel movement and the time needed for the patient to sit on a char and to ambulate. After patients' extubation and until day four, a surgeon of our department asked them twice daily to mark on the VAS the severity of the sternotomy pain. Patients also were asked of the occurrence of pruritus, nausea and vomiting by the same surgeon and all answers were noted. The time intervals from arriving at the ICU to discharge from ICU and discharge from the hospital were assessed as usual according to the department's standardized protocol for all patients undergoing cardiac surgery.

## Results

A total of 79 adult patients were enrolled in this study. However, three patients were excluded because they required prolonged intubation, more than two days. The postoperative course was uncomplicated for the rest 76 patients. During the first four postoperative days, the patients who received continuous subcutaneous anesthetic infusion experienced far less sternotomy pain than the rest of the departments' patients who were treated with opioid infusions in the usual way. Of the 76 patients, 57 did not need any supplemental opioid iv infusions and the rest needed only small infrequent doses, compared with the rest of the patients. All cases of significant pain complaints were checked with continuous ECG monitoring and troponine tests to rule out ischemic causes.

All 76 patients cooperated very well with the VAS and the results were excellent. The VAS rates were between 0 and 3 with a mean overall VAS score of 1,4. Moreover, the subjective pain estimation on behalf of the patients was equally good. (table 2) None of the patients complained of a postoperative sternotomy pain severe enough to disturb easy breathing and movement in any way. Each patient's primary nurse, along with filling the above mentioned form, also noted whether he/she believed that the patient had usual pain levels or improved pain control. The results were that 64% of patients didn't experience any significant pain at all, 29% experienced improved pain compared with the other cardiac surgical patients and only 7% of the patients enrolled in this study experienced the "usual" pain.

**Table 2:** VAS score in the study group

Patients	VAS score
17	0
29	1
24	2
6	3
0	Above 3

There was no drug toxicity identified in any of the 76 patients neither were any concerns about it during the overall postoperative course. There were no wound infections or wound healing complications in any of the patients in the study. Also, there were no complications related to placement or removal of the catheters. None of the patients experienced any discomfort at all related to the catheters or the catheter removal.

The postoperative length of hospital stay for the patients received catheter delivered analgesia was reduced. There was a difference in extubation time between the ropivacaine group and patients treated for pain with opioids (control group). A difference was also noted in the time needed for the first bowel movement. It was also obvious that patients enrolled in this study spent less time in the ICU, were able to get sit on the chair and get out of the bed sooner than the control group. (table 3). The mean length of stay (LOS) for the patients enrolled in this study was 5,8 days, with a range of 4 to 9 days. The usual mean LOS for cardiac surgical patients in our department is 6,7 days. All the above measured parameters are summarized in the table 2.

**Table 3:** Recovery times after surgery

	Ropivacaine group	Control group
Time to extubation(h)	6 (+/-2)	9 (+/-2)
First bowel movement(h)	4,3	7,4
ICU stay(h)	11,7	15,4
Time to sit on chair(h)	25,2	27,1
Time to walk(h)	38,4	54,7
LOS(d)	5,8	6,7

The difference in the mean LOS is statically significant and expresses the quicker discharge from the ICU and the hospital for the patients who received continuous subcutaneous catheter derived analgesia for their median sternotomy incisions.

Of note, none of the 76 patients had any pulmonary complications.

## Discussion

The effective management of pain is of paramount importance to both patients and doctors. This is particularly true to surgical patients. The most important source of postoperative pain is the surgical incision. There is increasing evidence that improved postoperative pain management, especially after major operations, has significant physiologic benefits. Surgical patients who can breathe easily and mobilize shortly after the operation enjoy much lesser respiratory complications, namely atelectasis and infections. On the other hand, mortality and morbidity after major operations are related at least partly to the pathophysiologic response to the major injury sustained by the surgical incision and to postoperative complications related to pain. Postoperative pain and the subsequent use of opioid analgesics to alleviate it is a major drawback to the uncomplicated postoperative recovery.

Postoperative pain after major procedures is mostly dealt with by ICU personnel and alleviated with use of opioid analgesics. This is mostly true for thoracic and abdominal operations during which huge muscle masses are incised or split apart [5]. However, opioid use can increase adverse effects and contribute to delays in postoperative recovery. Opioids can cause respiratory depression or even apnea, both of which lead to delays in weaning from the ventilator. Also, they can lead to nausea and vomiting and to subsequent aspiration chemical pneumonia. Moreover, ileus or constipation can prolong just by themselves the ICU stay of the patient. Jaundice is another dreadful side effect, caused by biliary spasm. Finally, opioids can cause ureteral or cystic spasm and lead to urinary retention. These adverse effects of opioid use mostly affect the immediate postoperative course of the surgical patient. It is obvious that the use of opioid-sparing analgesic techniques that can improve postoperative pain control with less or even without opioid medication might facilitate the recovery process and rehabilitation of surgical patients. However, no opioid-sparing analgesic regimen has been effective so far [2,4,6].

In cardiac surgery, most operations are performed through a median sternotomy. The current trend in postoperative management of cardiac patients is directed to early extubation and to fast-tracking of patients out of the ICU and subsequently the hospital. However, there have been no advances in methods to improve the control of pain after median sternotomy. Pain in cardiac ICUs is still treated with iv opioid analgesics. These agents are usually sufficient enough to relieve patients from postoperative pain and dismobility. However, opioids do have well known and above mentioned adverse effects. This can be a major setback to the well renounced fast-tracking postoperative cardiac surgical protocol. Cardiac surgical patients need to remain calm, breath with ease and get out of bed as soon as possible. The cardiac ICU personnel are fully aware of that and use opioid analgesics in a free manner to promote it. However, their side effects can undermine this fast-tracking protocol and prolong ICU stay of cardiac surgical patients.

Several alternative methods have been proposed instead of the traditional opioid use. NSAIDS were implied alone or combined with opioid administration but failed to show any particular benefit over opioids alone [6]. The so called combined treatment, implicating parasternal blockade together with intravenous opioids did not gain popularity mostly because of the short duration of act of the parasternal block [2,4,5]. On the other hand, there is a resurgence of interest in the use of continuous administration of local anaesthetics directly on the surgical wound in several surgical specialties. Regional infusion of ropivacaine has been successful in elective cesarean delivery as well as in colon and rectal surgery and after orthopaedic operations [7,8]. However, this method of analgesia has been mostly successful after thoracic operations performed with standard posterolateral thoracotomy. This highly painful incision has been successfully alleviated with single shots of continuous infusion of ropivacaine or bupivacaine directly in the intrapleural space. All these studies and clinical trials have proven the efficacy and safety of this method of regional analgesia and most of all the success in freedom from iv opioids.

Considering all the above facts we decided to proceed to a clinical trial implying direct and continuous infusion of a long acting topical anaesthetic agent directly on the sternotomy wound after cardiac operations. The nature of different cardiac procedures was not of importance because the postoperative pain is currently indifferently treated with opioids and the method of continuous anaesthetic infusion was examined to be efficient in every cardiac procedure. The severity of the operation and the preoperative condition of the patients were not considered drawbacks because this method has been already thoroughly tested in other surgical specialties as mentioned and proved generally safe.

The results were complimentary of the method of continuous infusion of local anaesthetics. This study clearly demonstrates that the use of ropivacaine as a long acting local anaesthetic delivered directly in the subcutaneous tissue of the median sternotomy incision can reduce or even negate the use of intravenous opioids after major cardiac procedures. The use of the VAS as an objective manner of testing postoperative pain proved the efficacy of this method. Moreover, nurses reported minimal opioid use. In fact most patients did not need any opioids at all. The relief of pain reported by nurses in charge of each patient was also significant. The patients implied in this study were free of disabling pain and the adverse effects resulting from it. All of them were able to easily breathe their way out of the ventilator and off the bed. None developed respiratory complications of any manner.

The subcutaneous catheters proved easy to use and remove. There were no infections in all patients enrolled in the study. The catheters did not cause discomfort in any way. Surgeons were able to perform wound dressing changes easily while the catheters were in place and the nursing personnel did not have any difficulty dealing with the elastomeric pump or the device as a whole. In fact, the pump was filled in the operation room and followed the patient thereafter as a unit until it was removed on day four. Thus there was no addition to the usual patient care on behalf of the nursing personnel.

Finally, the LOS was considerably reduced. This result matches the fast-tracking protocol widely accepted by cardiac surgeons and anaesthetists for cardiac surgical patients and clearly points the way traditionally opioid pain alleviation adversely affects this protocol.

In conclusion, the continuous infusion of ropivacaine directly on the sternotomy wound with specially designed subcutaneous catheters has been proved effective in reducing postoperative pain in cardiac operations of most kinds. This method also improved many patient outcome variables especially time to ambulation and LOS and most of all patient satisfaction with their pain management. This non-opioid analgesic method facilitates the fast-tracking protocol after open heart surgery.

## Authors' contributions

IK placed most of the catheters, conducted the study protocol and drafted the manuscript

MA was the primary surgeon in many cases and placed most of the catheters

AD was the anaesthesiologist and prepared the Ropivacaine solutions

VP assisted in most operations and managed the postoperative follow up of the patients

NT assisted in most operations and managed the postoperative follow up of the patients

CC is the head of the department and was the primary surgeon in many cases
